# Functional and clinical significance of ROR1 in lung adenocarcinoma

**DOI:** 10.1186/s12885-020-07587-6

**Published:** 2020-11-10

**Authors:** Schiavone Giovanna, Epistolio Samantha, Martin Vittoria, Molinari Francesca, Barizzi Jessica, Mazzucchelli Luca, Frattini Milo, Wannesson Luciano

**Affiliations:** 1 Istituto Oncologico della Svizzera Italiana, Via Ospedale, 6500 Bellinzona, Switzerland; 2 Istituto Cantonale di Patologia, Via in Selva 24, 6600 Locarno, Switzerland

**Keywords:** Non-small cell lung cancer, ROR1, Targeted therapies, Overall survival, EGFR

## Abstract

**Background:**

Receptor tyrosine kinase-like orphan receptor 1 (ROR1) is normally detectable in embryonic tissues and absent in adult tissues. ROR1 was shown to inhibit apoptosis, potentiate EGFR signaling and reported to be overexpressed and associated with poor prognosis in several tumor models. This study aimed to assess the expression of ROR1 in lung adenocarcinoma (AC) patients.

**Methods:**

We analyzed ROR1 expression by quantitative real-time PCR (qRT-PCR) in 56 histologically confirmed lung AC, stage I to IV, in addition we evaluated its association with TTF-1 (thyroid transcription factor-1) expression and the main molecular alterations involved in lung cancerogenesis.

**Results:**

ROR1 overexpression was observed in 28.6% of the entire cohort, using a cut-off of 1, or in 51.8% of the cases using the median value as threshold. Among patients without any genetic alteration, ROR1 overexpression was observed in 34.8% considering a cut-off of 1 and 52.2% considering the median value. The distribution of ROR1 was homogeneous among the different molecular categories: we found no association of ROR1 expression and the presence of gene mutations/rearrangements or the expression of TTF-1.

**Conclusions:**

ROR1 overexpression could constitute a potential therapeutic target because altered in a consistent number of lung AC, especially in cases without druggable genetic alterations. ROR1 expression is independent of classical lung cancer molecular alterations and not correlated, in a Caucasian cohort, to TTF-1 expression.

## Background

Despite several major achievements in the field of targeted and immune treatments for advanced non-small cell lung cancer (NSCLC), especially for the adenocarcinoma (AC) subgroup, this disease still represents a leading cause of death in Europe and worldwide [1]. At a molecular level, lung AC has been extensively characterized, but the enormous body of studies has led to the identification of only a subgroup of patients (cumulatively encompassing no more than 25% of cases) who have a molecular profile favorable to the efficacy of targeted therapies (i.e.: those carrying either EGFR mutations, or ALK, ROS1 or RET gene rearrangements) [2, 3]. Therefore, the identification of new molecular alterations and the development and application of related targeted strategies is essential to improve the prognosis of this disease.

The receptor tyrosine kinase-like orphan receptor 1 (ROR1) is an oncofoetal glycoprotein involved in differentiation, proliferation, migration and survival during the intrauterine development. ROR1 belongs to the ROR receptor tyrosine kinase family, the only other known member of which is ROR2, with a 58% amino acid sequence coincidence. The structure of human ROR1 comprises one FZ (frizzled) domain, one Ig-like (immunoglobulin-like) C2-type domain, one kringle domain and one protein kinase domain [4, 5]. ROR1 is normally expressed at high levels during development, becoming repressed in adult tissues. However, a low level of ROR1 expression is seen in adipose tissue and, to a lesser degree, in pancreas, lung and a subset of B cell leukemia [6, 7].

Interestingly, ROR1 may be re-induced during adult carcinogenesis. The expression of ROR1 was reported in numerous blood and solid malignancies, and appears to be involved in the inhibition of apoptosis [8]. In particular, silencing of ROR1 in NSCLC cells disrupts their ability to escape anoikis and anchorage-dependent programmed cell death, and shows decreased primary tumor growth when the cells are transplanted into nude mice. ROR1 seems to induce cell survival through at least two different mechanisms, one is mediated by the interaction with EGFR-Erb-B3 via the PI3K pathway, and the other one is dependent on its kinase activity via the c-SRC pathway [9]. Moreover, a recent study reports that ROR1 enhances lung adenocarcinoma growth by activating the Akt/GSK-3α/β/mTOR signaling cascade [10].

The observations of low or null ROR1 expression levels in normal adult tissues and its high expression levels in several cancer types led investigators to examine a potential functional advantage to cancer development and growth conferred by ROR1 and to explore the use of therapies against ROR1, that should be specific in cancer cells [11–15]. Yamaguchi and colleagues suggested that TTF-1 (thyroid transcription factor-1), a lineage-survival oncogene often expressed in lung AC, induces ROR1 expression, favoring by this mean a pro-survival PI3K-AKT activity, and opposing to the pro-apoptotic p38 signaling. They demonstrated that ROR1 knockdown inhibited lung AC cell lines, proposing that this receptor could represent a valuable therapeutic target in lung cancer patients [16]. Various putative approaches targeting ROR1 have been developed, such as blockage of its tyrosine kinase activity, the use of ROR1 as a surface target of monoclonal antibodies (MoAbs), [17] the use of MoAb-toxin conjugates (immunotoxins) or via chimeric antigen-receptor T-cells (CAR T-cell) [18].

Based on the observations described above, we hypothesized that ROR1 could be overexpressed in a significant proportion of lung ACs and potentially defines a subgroup of patients eligible for ROR1-targeted therapies. In addition, we aimed to assess the role of TTF-1 in the induction of ROR1 expression in human tissue samples. Finally, we looked for correlations of ROR1 and the main clinical and molecular features. .

## Methods

We investigated 56 lung adenocarcinoma specimens from the database of the Oncology Institute of Southern Switzerland (Bellinzona, Switzerland) which had enough tissue to perform RNA extraction for the evaluation of ROR1 expression by real-time PCR and to investigate TTF-1 expression, and EGFR, KRAS, BRAF, PIK3CA, HER2, ALK, ROS1 genetic aberrations. We subsequently investigated the association of ROR1 expression and molecular alterations in the whole, in the advanced and in the localized-disease populations. Finally, we analyzed the OS of patients according to ROR1 and TTF-1 expression, and to EGFR and KRAS mutations, since the number of patients with ALK, BRAF, PIK3CA, HER2, and ROS1 alterations was too small to allow a survival analysis.

Patients were ≥ 18 years old and had a histologically confirmed diagnosis of lung AC, stage I to IV as classified according to the seventh edition of the Cancer Staging Manual of the American Joint Committee on Cancer [19]. In our cohort, 7.1% of the patients had an advanced or metastatic disease and were treated with a palliative intention, while the vast majority (92.9%) presented localized disease and was treated with a curative intent.

### Determination of mutational status of EGFR, KRAS, BRAF, HER2 and PIK3CA by direct sequencing

Six 7-μm thick sections of formalin-fixed paraffin-embedded (FFPE) tissue containing at least 70% tumor cells (after manual microdissection) were cut for direct sequencing (DS) analyses. Genomic DNA was extracted using the QIAamp Mini kit (QIAGEN, Chatsworth, CA, USA) according to the manufacturer’s instructions. We searched for point mutations, deletions or insertions in EGFR exons 18–21, in KRAS exon 2, in BRAF exons 11 and 15, in PIK3CA exons 9 and 20, and in HER2 exon 20. The DS approach was based on the Sanger method, performed using a 3130 Genetic Analyzer (Applied Biosystems, Foster City, CA, USA), and the results were analyzed with the corresponding software (Sequencing Navigator, Applied Biosystems). Each sequencing reaction was performed at least twice starting from independent PCR reactions in order to confirm the mutation [20].

### ALK and ROS1 FISH analyses

FISH was performed using LSI ALK Dual Color Break Apart Probe (Abbott Molecular AG, Baar, Switzerland) and SPEC ROS1 Dual Color Break Apart Probe (Zytovision, Bremerhaven, Germany) on 4-μm thick FFPE sections, treated by VP2000 Instrument (Abbott Molecular AG, Baar, Switzerland). ALK and ROS1 evaluations were performed following international recommendations and as previously published [21].

### TTF-1 immunohistochemistry

For the evaluation of TTF-1 expression by immunohistochemistry (IHC), a mouse MoAb against TTF-1 (M3575; Dako©, Glostrup, Denmark) was used on a Benchmark XT platform (Ventana-Roche, Tucson, AZ, USA). When tumor cells presented nuclear staining, the case was considered TTF-1-positive. Data indicating the intensity, distribution and percentage of positive cells were also collected.

### Determination of ROR1 expression by real-time PCR

RNA was extracted from two 10-μm thick FFPE tissue sections. For each patient, tumor (containing at least 70% of tumor cells) and paired adjacent normal specimens were analyzed. RNA extraction was performed using the RNeasy FFPE kit (QIAGEN) according to the manufacturer’s instructions. For each specimen, 500 ng of RNA was retro transcribed into complementary DNA (cDNA) using the Superscript Vilo Mastermix III (Invitrogen, Carlsbad, CA, USA). ROR1 expression was evaluated by assays using a TaqMan fluorescent probe (Applied Biosystems) that recognizes the target gene (i.e., ROR1) and a TaqMan probe marked with a different fluorochrome that recognizes a reference gene (i.e., the RN18S1 housekeeping gene which encodes the 18S rRNA). For analysis of each specimen, 100 ng of retro transcribed RNA were used, and the amplification was performed in triplicate. The relative expression level was calculated with the Livak method, which standardizes the target gene to the reference gene in both tumor and normal tissues through the 2^[−ΔΔC(T)]^ formula. Gene expression levels were expressed as the fold change ± standard deviation. The specificity was determined by the analysis of the melting curves [22]. The real-time data were analyzed considering the threshold cycle in both cancer and normal tissues for each sample. ΔΔCt is the difference between the sample ΔCt and the control ΔCt. Sample ΔCt is the difference between the Ct of the target gene (ROR1) and the Ct of the reference gene in tumor tissue. Control ΔCt is the difference between the Ct of the target gene (ROR1) and the Ct of the reference gene in normal tissue. The cut-off value (r) for ROR1 overexpression was set-up at 1: cases showing r > 1 values were considered as ROR1 overexpressed, cases showing r ≤ 1 values were considered as having a normal expression of ROR1. We fixed 1 as cut-off because in literature a standard value to follow for the evaluation of ROR1 expression is not reported. We decided the threshold of r = 1 because the calculations of the Livak value generally are equal to 1 when the expression of a gene is comparable between normal and tumor tissues of the same patient. Furthermore, in agreement with another work of our group, we decided to subdivide the cohort on the basis of the median value [23].

### Statistical considerations

Demographic data and baseline characteristics of patients and disease were analyzed with standard summary statistics (mean SD and range for continuous data, relative and absolute frequencies for categorical data). Relationship of selected biomarkers with baseline factors and other biomarkers were analyzed by mean of a χ^2^ test. Time to event analysis was described by Kaplan Meier approach and the association with baseline characteristic was analyzed by proportional hazard model. For biomarker based on continuous scales, median and selected cut-off values were used.

## Results

**Fig. 1:**
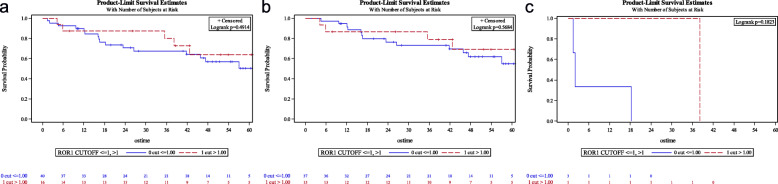
**a** Overall survival (OS): ROR1 by cut-off in the general population; **b** OS by ROR1 in localized disease population (cut-off); **c** OS by ROR1 in advanced disease population (cut-off)

**Fig. 2:**
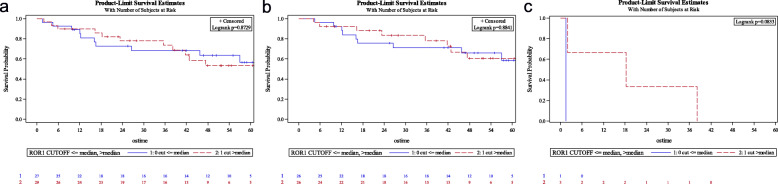
**a** Overall survival (OS) by median ROR1 expression; **b** OS by median ROR1 in localized disease population; **c** OS by median ROR1 in advanced disease population

Among the 56 lung adenocarcinoma samples, qRT-PCR showed ROR1 overexpression in 16 cases (28.6%) considering a cut-off value r = 1, while accounting for the median, patients with ROR1 overexpression were 29 (51.8%). The level of ROR1 overexpression, assessed both by the cut-off value (r > 1 vs r ≤ 1) (Fig. 1a, b and c) and the median one (over and below the median value) (Fig. 2a, b and c) was not associated with overall survival (OS) and this observation was independent of the tumor stage.

Concerning the main alterations clinically relevant in lung cancers, about 9% of patients had an AC harboring a classical EGFR mutation (exon 19 deletions or the exon 21 point mutation L858R). One patient presented EGFR mutation and ALK rearrangement simultaneously, while no patients showed an ALK rearrangement alone. A KRAS mutation was found in 46.4% of the patient population, constituting the most frequent genetic aberration. BRAF and HER2 mutations were found in one patient each, while no cases with ROS1 gene rearrangements were observed. As most of the tumor samples were initially evaluated in a pre-immunotherapy period, information on PD-L1 expression was not available and could not be evaluated prospectively due to insufficient tissue availability. The baseline patients’ and disease characteristics are summarized in Table 1.

**Table 1:** Patient’s characteristics

Patient’s Characteristics	no. (%)
**Sex**	
Male	26/56 (46.4%)
Female	30/56 (53.6%)
**Race**	
Caucasian	56/56 (100%)
**Stage of disease at diagnosis**	
I/II/IIIA	52/56 (92.9%)
IIIB/IV	4/56 (7.1%)
**Histologic Type**	
Adenocarcinoma	56/56 (100%)
**Mutation present**	
ROR1	16/56 (28.6%)
EGFR	5/56 (8.9%)
KRAS	26/56 (46.4%)
ALK	1/9 (11.1%)
BRAF	1/54 (1.9%)
HER2	1/55 (1.8%)
TTF-1	45/56 (80.3%)
ROS1	0/45 (0%)

Description of the clinicopathological features and of the mutational characterization of the cohort included in this study. Patient’s characteristics subgroups are reported in bold. Abbreviations: *no* number.

Among our patients without a genetic alteration, we found ROR1 overexpression in 34.8 and 52.2%, defined by r = 1 and a value over the median level of expression, respectively.

We also analyzed potential correlations between OS and ROR1 stratifying by the mutational status of the main markers involved in lung carcinogenesis and disease stage. In particular, we focused the attention on EGFR and KRAS mutations, because the alterations in the other genes were too rare. The distribution of ROR1 was homogeneous among the different mutational categories; in particular, we found no association of ROR1 expression and the presence of EGFR or KRAS mutations. Moreover, we did not find any correlation between ROR1 and TTF-1 expressions, unlike suggested by other authors in an Asian population [24] (Table 2).

**Table 2:** Association of ROR1 with: TTF-1 expression; EGFR, KRAS mutations by median and cut-off

Overall population		ROR1 expression					
		≤ median	>median	*p* value	cut-off ≤1	cut-off > 1	*p* value
EGFR	wt	25	26	**0.7027**	37	14	**0.5569**
	mut	2	3		3	2	
KRAS	wt	14	16	**0.8051**	20	10	**0.4011**
	mut	13	13		20	6	
TTF-1	neg	6	5	**0.6422**	8	3	**0.9161**
	pos	21	24		32	13	
**Localized disease**		ROR1 expression					
		≤ median	>median	p value	cut-off ≤1	cut-off > 1	p value
EGFR	wt	24	23	**0.6413**	34	13	**0.5663**
	mut	2	3		3	2	
KRAS	wt	13	14	**0.7834**	17	10	**0.1797**
	mut	13	12		20	5	
TTF-1	neg	6	5	**0.7367**	8	3	**0.8978**
	pos	20	21		29	12	
**Advanced disease**		ROR1 expression					
		≤ median	>median	p value	cut-off ≤1	cut-off > 1	p value
EGFR	wt	1	3	**0**	3	1	**0**
	mut	0	0		0	0	
KRAS	wt	1	2	**0.5637**	3	0	**0.0833**
	mut	0	1		0	1	
TTF-1	neg	0	0	**0**	0	0	**0**
	pos	1	3		3	1	

Statistical evaluation of ROR1 expression distribution, estimated by the median and the cut-off (equal to 1), among the analyzed molecular markers: EGFR, KRAS mutations; TTF-1 expression. The *p* values results and the severity of the disease are reported in bold

Parallel statistical evaluations demonstrated that, in our patient cohort, there were no differences in OS of patients with EGFR mutated lung adenocarcinoma versus those with EGFR wild-type tumors, irrespective of the stage (*p* = 0.9875). Among patients with advanced stage, the presence of EGFR-mutation had a non-significant trend to a better OS comparing to patients with localized disease (*p* = 0.0703). For patients with localized disease, the OS curves of the EGFR wild-type and mutated populations were superimposable (*p* = 0.8529).

Similarly, the presence or absence of a KRAS mutation did not have a correlation with OS (*p* = 0.8160). Patients with KRAS wild-type and localized disease were found to have a non-significant trend to a better OS (*p* = 0.0767); however, in those with advanced disease, the OS was similar regardless of the presence or absence of a KRAS mutation (*p* = 0.8887).

TTF-1 was found to be consistently expressed in 80,3% of our cohort but was not associated with OS neither in general (*p* = 0.1619) nor in the localized (*p* = 0.3601) or advanced disease populations (*p* = 0.1707). TTF-1 expression was not correlated with ROR1 expression.

## Discussion

In recent years, relevant improvements in the treatment of lung AC were achieved, especially by virtue of the introduction of tailored treatment acting against specific molecular targets and immunotherapy with checkpoint inhibitors [25–28]. Nevertheless, the 5-year OS rates remain unsatisfactory for a significant number of patients without a druggable mutation or low/negative PD-L1 expression. The identification of novel predictive and prognostic biomarkers as well as potential targets for novel treatment strategies is therefore of great importance.

ROR1 is an embryonic protein which plays a fundamental role in cardiorespiratory, neurological and skeleton development, but its expression is very rare in adult tissues [8]. In the other hand, some studies reported that ROR1 could be overexpressed by several hematological and solid malignancies [15, 29–31]. The specific expression of ROR1 by tumor cells and its absence in normal tissues makes of this receptor an ideal theoretical target for cancer treatment. The relevance of ROR1 and its therapeutic role has been recently explored in ovarian cancer. Indeed, it has been demonstrated that ROR1 has an important therapeutic role in such a neoplastic disease because glucocorticoids anti-inflammatory agents (generally administred as adjuvant to chemotherapy) may induce chemoresistance by promoting ROR1-mediated stemness [32].

Preliminary studies on ROR1 expression in human lung AC either included a small number of patients or were conducted on cell lines. Zhang and colleagues studied 29 lung AC patients and showed that 59% presented a strong expression of ROR1 by IHC [14]. Karachaliou and co-workers assessed ROR1 mRNA expression in 27 NSCLC patients with the EGFR-T790M mutation, showing that high ROR1 expression is associated with a significantly shorter progression-free survival in erlotinib-treated patients, but not in chemotherapy-treated patients [33]. The results of a study by Zheng and colleagues revealed that ROR1 protein expression was significantly higher in lung AC tissue than in the adjacent normal tissue. Furthermore, patients with advanced disease showed higher levels of ROR1 expression, and this study revealed an association between high ROR1 expression and worse OS [24].

In the present study, we examined the mRNA expression of ROR1 in 56 lung adenocarcinoma patients by qRT-PCR. We decided to use an objective methodology (i.e.: qRT-PCR) because very often the evaluation of IHC staining is difficult and can vary depending on time and type of fixation. Furthermore, there is no a specific and widely approved antibody for IHC, the procedure for the evaluation of ROR1 protein expression is not standardized and the results obtained using different antibodies can give not reproducible results, as clearly demonstrated by several contributions, including one where three different antibodies, evaluated with three different instruments, were compared for assessing the expression of another receptor tyrosine kinase like EGFR (located in plasma membrane, as ROR1). An open discussion topic is represented by the cut-off value for the definition of expression of genes by qRT-PCR, due to the lack of consensus in the literature. We hence decided to use two different definitions: the median value (that is cohort-dependent but represents the wider approach in the literature) and the value of *r* = 1, that indicates a clear overexpression (if > 1) with respect to a normal expression (≤ 1). In our cohort, we found relevant rates of ROR1 mRNA overexpression even when the cut-off *r* = 1 was used (28.6%). This proportion is increased when we focused on patients without any mutation in the genes traditionally involved in lung carcinogenesis such as EGFR, KRAS, BRAF, HER2, ALK, ROS1 (34.8 and 52.2% when we applied the cut-off of *r* = 1 or of the median value, respectively), supporting the hypothesis that ROR1 could constitute a potential tumor driver in a significant number of lung AC. To our best knowledge, this is the first assessment of ROR1 on a Caucasian cohort of patients. Unlike a previous report conducted on a cohort of Asian patients that suggested that ROR1 overexpression is induced by TTF-1, [16] we did not find any correlation between ROR1 mRNA levels and TTF-1 expression. Furthermore, in contrast to previously reports, [34–36] in our patient cohort TTF-1 expression was not prognostic for survival, although we acknowledge that this result could be biased by the low number of patients that we analyzed.

ROR1 expression was homogeneously distributed among the different tumor types, sorted by mutation status. We found, in fact, no correlation between expression of ROR1 and the presence of EGFR and KRAS mutations. Finally, we did not find any correlation between ROR1 expression (irrespectively of the cut-of value used) and survival.

A corollary of the analysis of ROR1 was the evaluation, in terms of prognostic factor, of EGFR or KRAS mutations. In our cohort, there were no differences in OS of patients with EGFR mutated lung AC versus those with EGFR wild-type tumors, irrespective of the stage of the disease. This result was probably due to a prevalence of resected cases over advanced, non-resected stages. Among our few patients with an advanced stage, EGFR-mutated disease had a non-significant trend to a better OS. For patients with localized disease, the OS curves were superimposable. This phenomenon may be explained by the fact that less resected patients with EGFR mutated disease undergo progression and eventually benefit from anti-EGFR TKI therapy.

## Conclusions

In conclusion, our study demonstrates that ROR1 overexpression in lung AC occurs in a significant number of patients, suggesting a potential role of ROR1 as a therapeutic target, especially for patients who cannot be treated with current targeted therapies due to the lack of molecular alterations in their cancers. Standardization of the assessment of ROR1 protein expression is still pending.

## Data Availability

The datasets used and analysed during the current study are available from the corresponding author upon request.

## Abbreviations

AC: Adenocarcinoma
CAR T-cell: Chimeric antigen-receptor T-cells
cDNA: Complementary DNA
DS: Direct sequencing
FFPE: Formalin-fixed paraffin-embedded
FZ: Frizzled domain
Ig-like: Immunoglobulin-like
IHC: Immunohistochemistry
MoAb: Monoclonal antibody
NSCLC: Non-small cell lung cancer
OS: Overall survival
qRT-PCR: Quantitative real-time PCR
ROR1: Receptor tyrosine kinase-like orphan receptor 1
TTF-1: Thyroid transcription factor-1
