# Correction to: Functional and clinical significance of ROR1 in lung adenocarcinoma

**DOI:** 10.1186/s12885-020-07704-5

**Published:** 2020-12-07

**Authors:** Giovanna Schiavone, Samantha Epistolio, Vittoria Martin, Francesca Molinari, Jessica Barizzi, Luca Mazzucchelli, Milo Frattini, Luciano Wannesson

**Affiliations:** 1 Istituto Oncologico della Svizzera Italiana, Via Ospedale, 6500 Bellinzona, Switzerland; 2 Istituto Cantonale di Patologia, Via in Selva 24, 6600 Locarno, Switzerland

**Correction to: BMC Cancer 20, 1085 (2020)**

**https://doi.org/10.1186/s12885-020-07587-6**

Following publication of the original article [1], the authors reported that the family names and the given names of the authors were interchanged. The names published in this correction article have been corrected and are as follows:

Giovanna Schiavone (given name, family name)

Samantha Epistolio

Vittoria Martin

Francesca Molinari

Jessica Barizzi

Luca Mazzucchelli

Milo Frattini

Luciano Wannesson

The original article [1] has been corrected.
